# P2×7 targeting inhibits growth of human mesothelioma

**DOI:** 10.18632/oncotarget.10430

**Published:** 2016-07-06

**Authors:** Francesca Amoroso, Erica Salaro, Simonetta Falzoni, Paola Chiozzi, Anna Lisa Giuliani, Giorgio Cavallesco, Pio Maniscalco, Andrea Puozzo, Ilaria Bononi, Fernanda Martini, Mauro Tognon, Francesco Di Virgilio

**Affiliations:** ^1^ Department of Morphology, Surgery and Experimental Medicine, University of Ferrara, Ferrara, Italy

**Keywords:** cancer, mesothelioma, purinergic signalling, extracellular ATP, P2×7

## Abstract

Malignant pleural mesothelioma (MPM) is an aggressive tumor refractory to anti-blastic therapy. MPM cells show several genetic and biochemical defects, e.g. overexpression of oncogenes, downregulation of onco-suppressor genes, dysregulation of microRNA, or alteration of intracellular Ca^2+^ homeostasis and of apoptosis. No information is as yet available on purinergic signalling in this tumor. Signalling via the P2×7 (P2RX7 or P2×7R) purinergic receptor is attracting increasing attention as a pathway involved in cancer cell death or proliferation. In this report we show that the P2×7R is expressed by three MPM cell lines established from MPM patients but not by mesothelial cells from healthy subjects (healthy mesothelial cells, HMCs). MPM cell proliferation was inhibited by *in vitro* incubation in the presence of selective P2×7R antagonists, as well as by stimulation with the P2×7R agonist BzATP. Systemic administration of the selective P2×7R blocker AZ10606120 inhibited *in vivo* growth of MPM tumors whether implanted subcutaneously (s.c.) or intraperitoneally (i.p.). Our findings suggest that the P2×7R might be a novel target for the therapy of mesothelioma.

## INTRODUCTION

Human malignant pleural mesothelioma (MPM) is a mesothelium-originated tumor most commonly involving the lungs, and more rarely the peritoneum and the vaginal membrane of the gonads. Its latency is long (15–40 years) and the prognosis very poor, with an overall survival time from diagnosis of less than one year. Extensive epidemiological data now demonstrate that the most important etiological factor is asbestos exposure. Rather intriguingly, there seems to be no correlation between length of exposure and risk of developing MPM [1]. There is no effective therapy for MPM, the commonly used treatment being pleuro-pneumectomy variably associated with neo-adjuvant and/or adjuvant chemotherapy, or radiotherapy and neo-adjuvant radiotherapy [2, 3]. A number of small Phase I/II trials using targeted therapy, i.e. inhibitors of vascular endothelial growth factor receptor (VEGFR), phosphatidylinositol 3-kinase/mammalian target of rapamycin (PI3K/mTOR), histone deacetylase and proteasome inhibitors are ongoing [4, 5, 6]. Gene therapy and immunotherapy are also actively investigated [7, 8]. Despite all these different therapeutic efforts, MPM is still a malignancy both difficult to diagnose and treat. Lack of effective treatments has spurred an active search for new therapeutic strategies to improve patients' quality of life and prolong survival.

During the last few years several studies have shown that plasma membrane receptors for extracellular nucleotides, i.e. the P2 receptors (P2Rs), promote inflammation, cancer cell growth and metastatic spreading [9, 10, 11, 12, 13, 14]. Among the P2Rs, a special role in cancer is played by the P2×7 receptor (P2RX7 or P2×7R). We and others have shown that the P2×7R supports tumor cell growth both *in vitro* and *in vivo*, [13, 11], affords a proliferative advantage under limiting growth condition and glucose deprivation [15, 16, 17], facilitates extracellular matrix invasion [9], and stimulates VEGF secretion [13]. Moreover, the PI3K/GSK3β/HIF-1α pathway was recently identified as a main pathway responsible for P2×7R-mediated growth stimulation in human neuroblastoma cells [11]. Finally, we showed that administration of P2×7R blockers has a strong growth-inhibitory effect in several transplanted tumors such as melanoma, colon carcinoma and human and mouse neuroblastoma [13, 11]. Interestingly, we also observed that systemic administration of P2×7R blockers was more affective in tumor-bearing immunocompetent versus *nude/nude* mice [11], suggesting that the anti-tumor effect required an active immune response.

Expression of ATP receptors, notably P2×7R, is thought to have profound effects on host-tumor interaction since there is now very solid proof that the tumor microenvironment (TME) is enriched in ATP [18, 19, 20]. In the TME this nucleotide may promote tumor growth (for example via the P2×7R) and at the same time enhance host defenses, because tumor infiltrating immune cells also express P2×7R and other nucleotide/nucleoside receptors [20]. An ATP-rich TME can be a double-edge sword since P2×7R overstimulation can also be a trigger of cytotoxicity [21], but in tumors this untoward effect is apparently mitigated by an as yet poorly understood mechanism that uncouples P2×7R from downhill pro-apoptotic pathways otherwise active in healthy cells [22].

While P2×7R expression and function in several human tumors is well documented [12, 23], no data are so far available in human MPM. Therefore, in this study we aimed at investigating P2×7R expression in healthy mesothelial and malignant mesothelioma cells, and the effect of P2×7R inhibition on *in vitro* and *in vivo* tumor growth.

Our data show that the P2×7R is expressed in MPM cells, while it is completely absent in mesothelial cells from healthy subjects. Immortalization of human mesothelial cells with the pSV3Neo plasmid expressing the Simian Virus 40 (SV40) T-antigen (Tag) (SV40-Tag cells) caused P2×7R upregulation. Cellular responses activated by the P2×7R agonist benzoyl ATP (BzATP) were barely detectable in all mesothelial cells types investigated. Growth rate of mesothelial cells *in vitro* (with the notable exception of the SV40-Tag cells) was slow. Nevertheless, we were able to clearly inhibit growth by treatment with P2×7R agonists or antagonists. Growth inhibition was especially associated to release of the cytoplasmic marker lactate dehydrogenase (LDH) in SV40-Tag cells and MPP89 cells. MSTO-211H and IST-MES2 were inoculated subcutaneously (s.c.), while MPP89 were inoculated intraperitoneally (i.p.) in immunocompromised, *nude/nude*, mice. In all these three models, P2×7R targeting strongly reduced tumor growth. This is the first study to our knowledge showing that P2×7R blockade effectively inhibits *in vivo* MPM tumor growth.

## RESULTS

### P2×7R expression and function in healthy human mesothelial cells and in MPM cells

**Figure 1:**
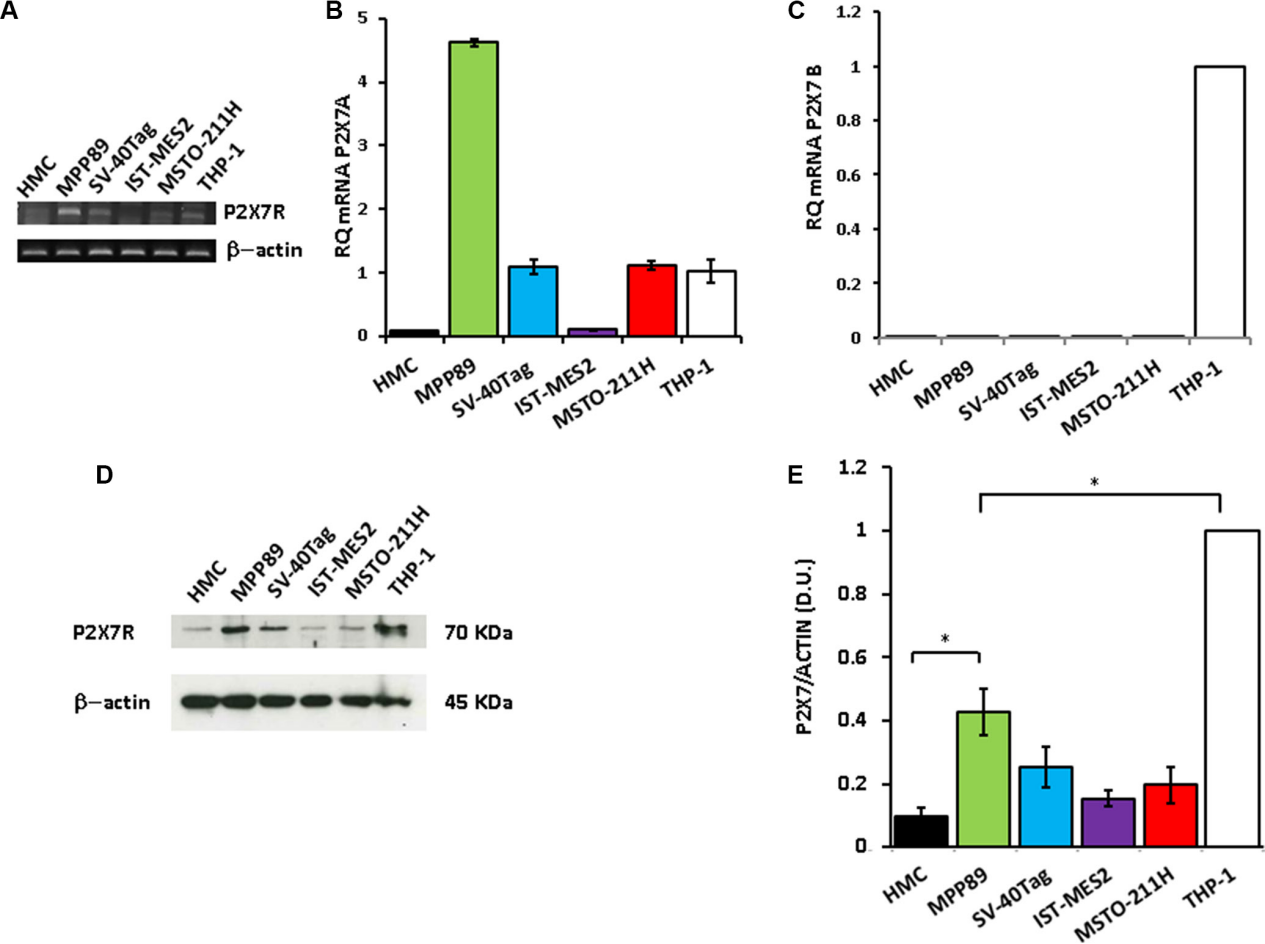
P2×7R expression in MPM cells P2XR7 expression was measured by RT-PCR (**A**), qRT-PCR (**B**), and Western blotting (**D**–**E**). (**C**) qPCR for P2×7RB is shown. PCR, qPCR and Western blotting were performed as described in Materials and Methods. β-Actin was used as loading control. ΔΔCt was determined by assuming THP-1 cells as reference sample and human β- actin as endogenous control. For densitometry (E), P2×7R protein bands were normalized on the β- actin band. B. *p* < 0.001 for MPP89 versus HMC; *p* < 0.01 for SV-40TAG, MSTO-211H and THP-1 versus HMC. E. *p* < 0.001 for MPP89, SV40-Tag, MSTO-211H and IST-MES2 versus THP-1. E. *p < 0.05. Data are averages + SE from triplicate samples.

P2×7R expression was investigated in healthy human mesothelial cells (HMCs), in three MPM cell lines (MPP89, IST-MES2 and MSTO-211H) and in human mesothelial cells immortalized by SV40-Tag transfection (SV40-Tag cells). In HMCs P2×7R was virtually absent whether by conventional (Figure 1A) or qRT-PCR (Figure 1B). In MPP89, MSTO-211H and SV40-Tag cells, P2×7R transcript expression was comparable to that of THP-1 cells, a human leukemic cell line known to express this receptor. IST-MES2 expressed 3-4 fold less, and barely detectable, P2×7R transcript. Conventional PCR showed that MPP89 cells have a high P2×7R transcript content (Figure 1A). This finding was quantitatively confirmed by RT-PCR showing that this MPM cell line has a three fold higher P2×7R transcript level than the other cell lines, THP-1 included (Figure 1B). We also analyzed by qRT-PCR expression of the other P2×7R main splice variant, the truncated isoform named P2×7B (P2×7RB). As shown in Figure 1C, the P2×7RB isoform was virtually absent in all mesothelial cell lines. The P2×7R protein was present at low levels in HMC, IST-MES2, MSTO-211H and SV40-Tag cells cells, while it was about 2 fold higher in MPP89 cells. However, even in MPP89 cells the level of P2×7R protein expression was about half of that of THP-1 cells (Figure 1D–1E). These findings show that MPM cells express P2×7R to a low level, and therefore we anticipated that P2×7R-dependent responses should also be very low.

**Figure 2:**
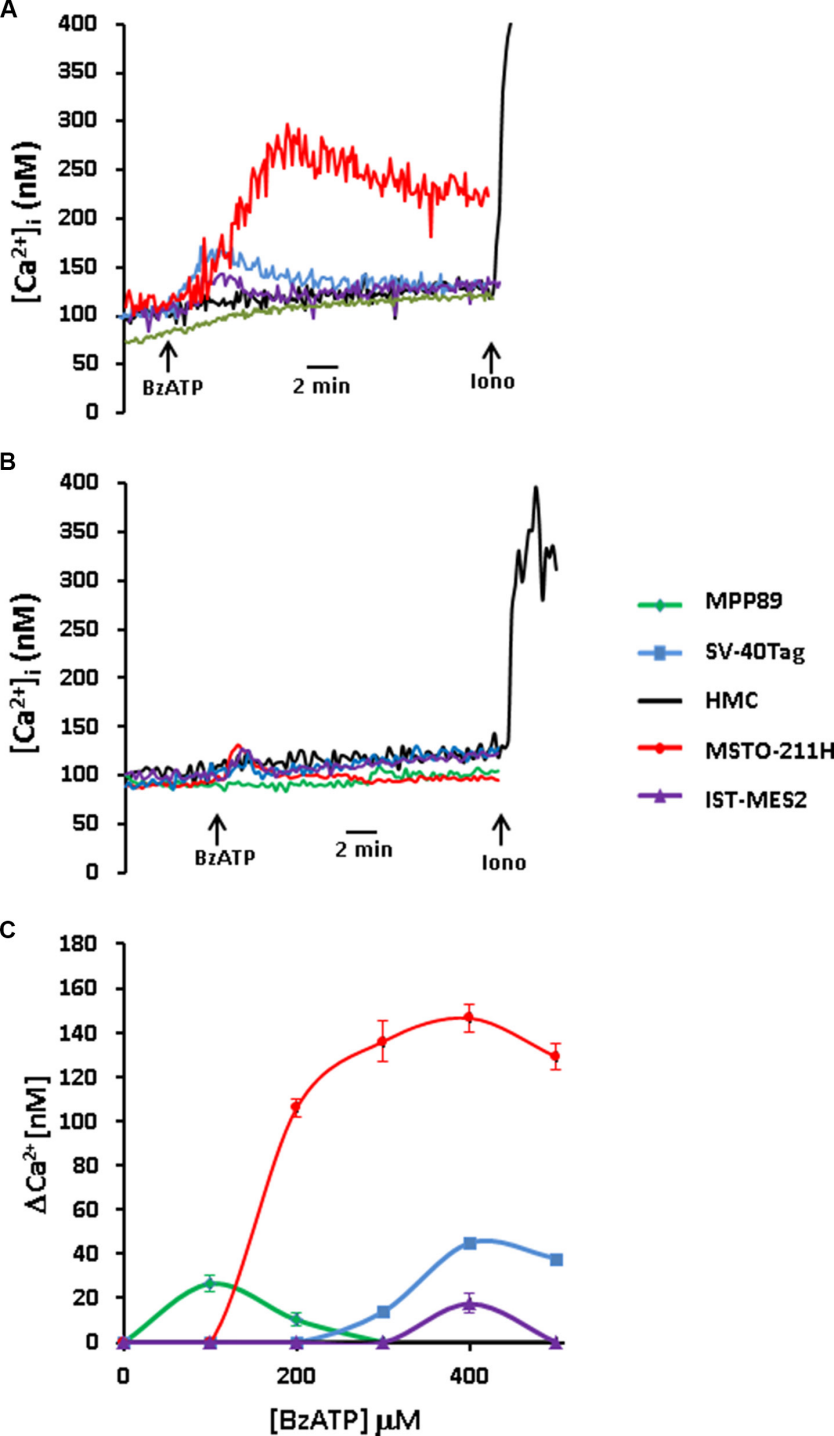
P2×7R-stimulated cytoplasmic Ca^2+^ increases in MPM cells are blocked by AZ10606120 Cytosolic Ca^2+^ was measured as described in Materials and Methods. (**A**) BzATP and ionomycin (iono) were added at the concentration of 300 and 1 μM, respectively. (**B**) Effect of AZ10606120 on the BzATP (300 μM)-stimulated Ca^2+^ increase. AZ10606120 was added to the cell suspension 5 min prior to BzATP addition. Ionomycin was 1 μM. (**C**) BzATP dose-response curve. Δ cytosolic Ca^2+^ increase (Δ [Ca^2+^]_i_) is the difference between peak and basal Ca^2+^ level. In the SV40-Tag curve error bars are not shown because they are smaller than the symbol. Data are averages ± SE of three to five independent determinations.

To verify this anticipation, we investigated two short term responses dependent on P2×7R stimulation: a) increase in the intracellular Ca^2+^ concentration, and b) permeabilization of the plasma membrane to high MW aqueous solutes (large pore opening), generally thought to be the functional signature of P2×7R. As shown in Figure 2, the semi-selective P2×7R agonist BzATP triggered a cytoplasmic Ca^2+^ increase in all cell lines, except HMCs, which were unresponsive throughout the whole range of BzATP concentrations tested. Ca^2+^ increases were rather small compared to those observed in other P2×7R-expressing cell types, but reflected the expected BzATP dose dependency, with a maximally stimulatory dose around 400 μM. The MMP89 cells were a relevant exception as BzATP concentration higher that 100 μM caused a strong inhibition of the Ca^2+^ influx. In all MPM cell lines the BzATP dose-dependency of the cytoplasmic Ca^2+^ increase was bell-shaped. This was due to progressive chelation of extracellular Ca^2+^ at increasing BzATP concentrations, as previously shown by us in several different cell types [24, 20]. On the basis of the level of P2×7R protein expression, we anticipated that MPP89 cells should undergo the largest Ca^2+^ rise, followed by the SV40-Tag cells. At variance from this anticipation, MSTO-211H cells showed a BzATP-stimulated Ca^2+^ rise 4-5 fold higher than that of MPP89 cells, suggesting that in these latter cells the P2×7R might be in part sequestered intracellularly and thus not fully accessible to BzATP stimulation on the plasma membrane. In the SV40-Tag cells P2×7R stimulation triggered a Ca^2+^ rise in the 30–40 nM range, about twice the Ca^2+^ increase measured in IST-MES2 cells (15–20 nM) (Figure 2C). The Ca^2+^ rise was almost completely obliterated by AZ10606120, except for a small and transient Ca^2+^ increase observed in MSTO-211H and SV-40Tag cells (Figure 2B). In all mesothelial cell types we were unable to detect any biochemical hints suggesting the opening of large conductance pore which is generally assumed to be the functional P2×7R signature, possibly because of its low expression levels in MPM. Lack of large pore opening might depend on the preferential expression of the defective P2×7RB splice variant, as described by Adinolfi et al. [25], but this was not the case as MPM expressed no P2×7RB, as shown in Figure 1C. We also analyzed the expression of other P2XRs and P2YRs, but only P2×4R, P2Y2R and P2Y11R were expressed albeit to a very low level (not shown).

**Figure 3:**
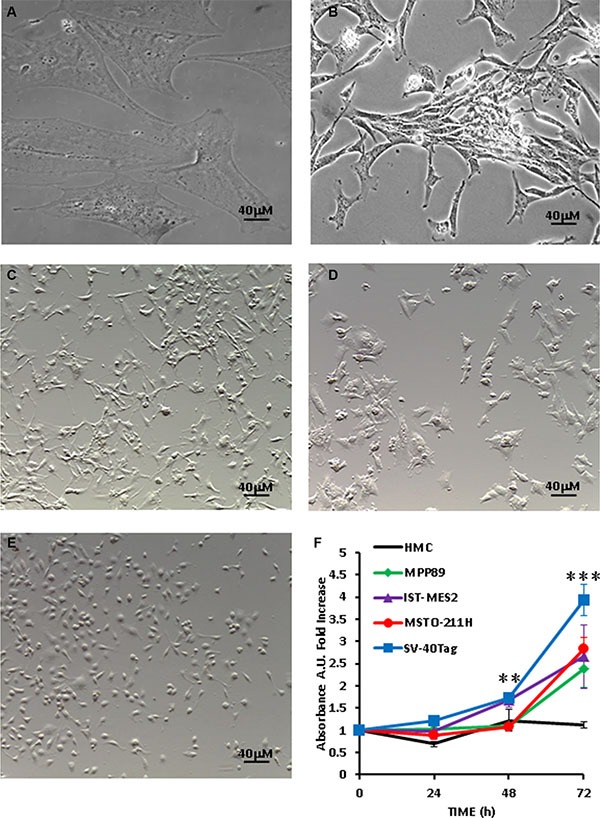
Growth rate and morphology of MPM cells (**A**) HMCs; (**B**) SV40-Tag cells; (**C**) MPP89 cells; (**D**) MSTO-211H cells; (**E**) IST-MES2 cells. (**F**) Growth kinetics of HMCs and MPM cells cultured in serum-free RPMI or serum-free DMEM-F12 (MPP89) medium for 72 h; cell number was assessed by measuring crystal violet uptake at 590 nm absorbance as described in Materials and Methods. Pictures were taken with a Leica phase contrast microscope as described in Materials and Methods.

We and others have shown that basal expression or low level stimulation of the P2×7R, as opposed to sustained pharmacological stimulation, drives cell growth, and accordingly P2×7R silencing or pharmacological blockade inhibits proliferation [15, 13, 26, 27]. Growth-stimulating effect of P2×7R is enhanced in low nutrient culture conditions [16, 17]. Figure 3F shows that in the absence of serum HMCs were fully growth-arrested, while on the contrary MPM cells retained their growth ability. It is worth noting that SV40-Tag cells showed a growth kinetic that was twice as fast as that of the other MPM cell lines. As shown in panels 3A–3E, the five different mesothelial cell lines used in this study showed striking morphological differences: HMC were thin, flattened and very large (over 40 μm in diameter and 100 μm in length), with a typical mesothelial shape; SV40-Tag were fibroblast-like and about 40 μm in length. The other three cell lines were rather heterogeneous in shape and size, being IST-MES2 the smallest (with a diameter between 10 and 15 μm). We next verified whether proliferation of MPM cells was affected by P2×7R targeting.

### P2×7R targeting inhibits MPM cell proliferation *in vitro*

**Figure 4:**
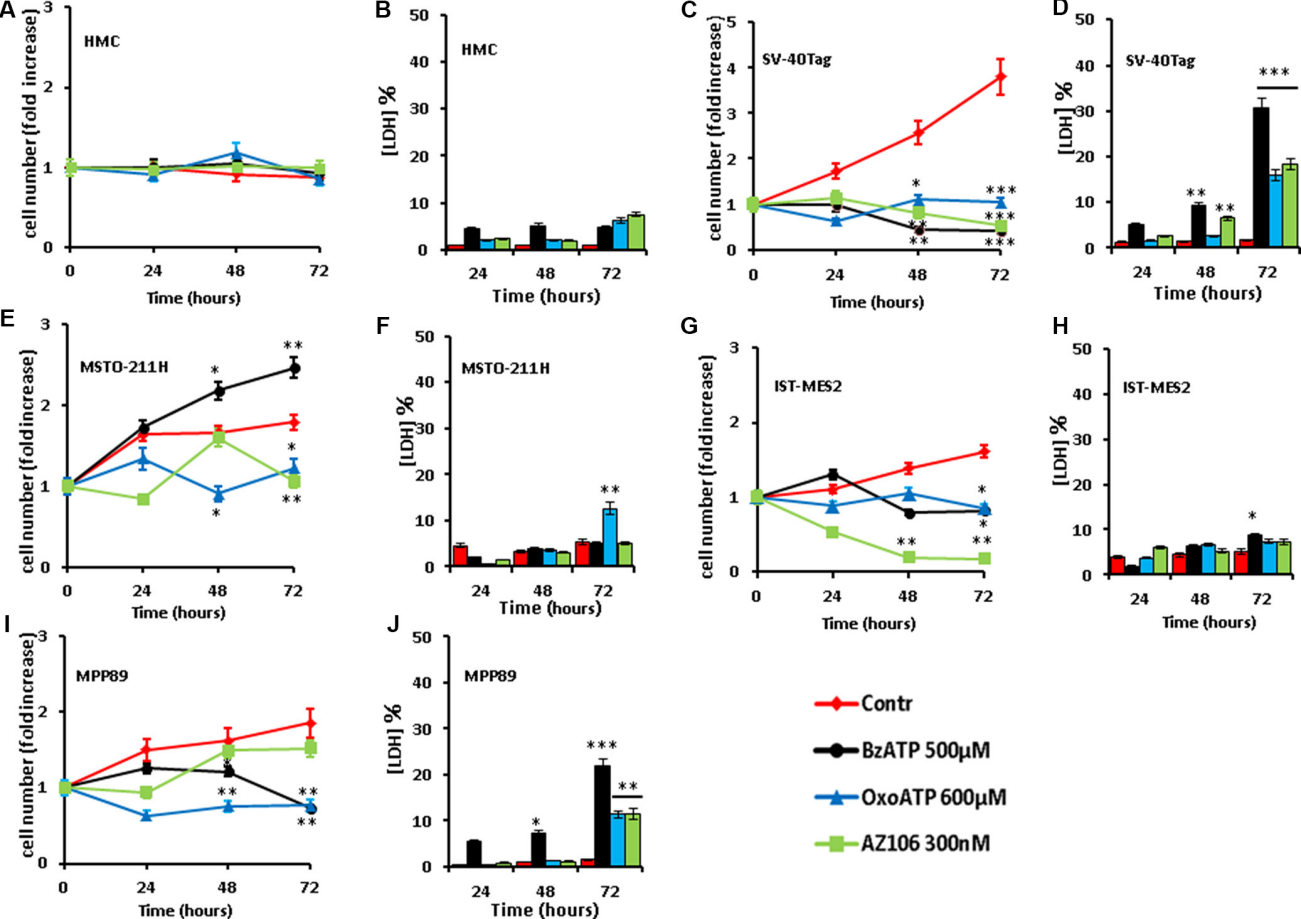
P2×7-targeting inhibits *in vitro* MPM cell growth HMCs and MPM cells were seeded in 12-well plates as described in Materials and Methods and incubated in serum-free medium for the indicated time in the absence or presence of the various agents. Samples run in parallel were used for measurement of cell growth (panels **A**, **C**, **E**, **G** and **I**) and lactic dehydrogenase (LDH) release (panels **B**, **D**, **F**, **H** and **J**). Data are averages ± SE from nine separate determinations from three different experiments. Statistical significance of treated samples versus controls: ***p < 0.001; **p < 0.01; *p < 0.05.

Responses of MPM cells to P2×7R activation or blockade were rather heterogenous. HMCs were insensitive to P2×7R targeting whether by agonists or antagonists (Figure 4A–4B). No significant decrease in cell number was observed in HMCs, nor lactic dehydrogenase (LDH) release as an index of P2×7R-dependent necrosis. At variance from HMCs, proliferation of SV40-Tag cells was sensitive to P2×7R modulation (Figure 4C–4D). The pharmacological ATP analogue BzATP strongly inhibited cell growth and caused a significant LDH release. P2XR7 blockade by the highly selective antagonist AZ10606120 or by the less specific covalent blocker oxidized ATP (oxoATP) inhibited proliferation and increased LDH release. MSTO-211H cell proliferation was fully blocked by oxoATP or by AZ10606120, but contrary to the other cell types BzATP showed growth-promoting activity (Figure 4E–4F). Of all the stimuli, only oxoATP triggered about 10% LDH release at the 72 h time point. IST-MES2 cell proliferation was blocked by P2×7R agonists or antagonists at all time points (Figure 4G–4H). In this cell type, AZ10606120 caused a severe reduction in cell number in the absence of significant LDH release, suggesting an apoptotic process. MPP89 cell proliferation was fully inhibited by BzATP or by oxoATP, while AZ10606120 had a negligible effect (Figure 4I–4J). BzATP, oxoATP and AZ10606120 had a small but significant cytotoxic effect in MPP89 cells. Despite a certain degree of heterogeneity, these results provide two relevant pieces of information: a) HMCs lack P2×7R and are fully growth arrested in the absence of serum, while MPM cells (especially SV-40-Tag) express P2×7R and retained growth ability under this condition of limited nutrient supply; b) P2×7R blockade inhibits MPM proliferation. While these data do not allow to conclude that P2×7R expression is the factor that supports growth of MPM or SV40-Tag cells in the absence of serum, the inhibitory effect of P2XR7 antagonists suggest that this receptor is involved. To gain more solid hints as to the role of P2×7R in MPM growth, we turned to the *in vivo* model.

### P2×7R targeting inhibits *in vivo* mesothelioma growth

**Figure 5:**
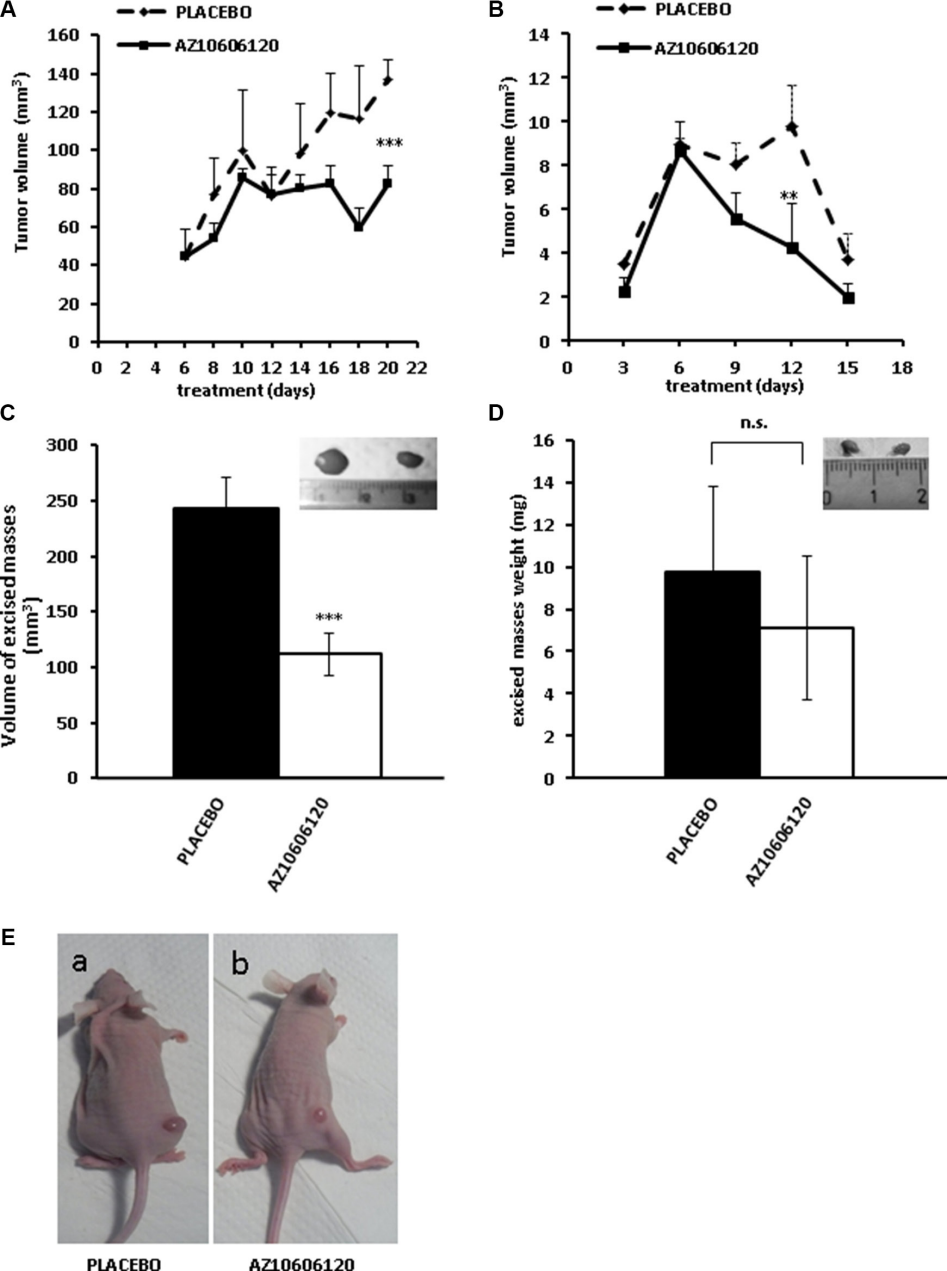
P2×7R-targeting inhibits growth of s.c. inoculated MPM cells 2 × 10^6^ MSTO-211H (panels **A**, **C**, **E**) or IST-MES2 (panels **B**, **D**) cells were s.c. inoculated into *nude/nude* mice as described in Materials and Methods. Tumors became palpable 4 d after inoculum with both MPM cell lines although the IST-MES2 mass was much smaller than the MSTO-211H mass. On post-inoculum d 6 AZ10606120 (0.7 mg/kg), or placebo (DMSO 0,05% in 100 μl PBS solution) were administered intra mass every two d for up to 20 d. Five (MSTO-211H) or 3 (IST-MES2) mice were used for AZ10606120 and placebo administration, respectively. A, B. *In vivo* growth kinetic. Data are averages ± SE. ***p < 0.001; **p < 0.01. B, D. Volume of excised tumors. *N* = 5 (MSTO-211H), or 3 (IST-MES2); ***p < 0.001; n.s., not significant. Tumor size was measured by caliper and volume calculated as described in Material and Methods. E. Pictures showing tumor size at post-inoculum d 20 in two MSTO-211H-injected mice respectively treated with placebo (**a**) or AZ10606120 (**b**). Insets in panels C and D show representative pictures of excised tumors from placebo (left)- or AZ10606120-treated (right) mice.

To assess the effect of P2×7R blockade on mesothelioma growth *in vivo*, *nude/nude* mice were subcutaneously (s.c.) inoculated with either MSTO-211H (Figure 5A, 5C, 5E) or IST-MES2 (Figure 5B–5D) cells and intra-mass (i.m.) injected with AZ10606120 or placebo (PBS + 0.05% DMSO). AZ-10606120 or placebo were injected every two d after tumor mass became first detectable (d six from tumor cell inoculation), and tumor mass was assessed by caliper measurement for a total of 20 (MSTO-211H) or 15 (IST-MES2) d. For the MSTO-211H inoculum, initial kinetic of tumor growth (from post-inoculum d 6 to d 10) was due both to the increase in cell number and to the tumor-associated edema (Figure 5A). After d 10, edema started to regress while the tumor mass continued its growth and bulged out of the skin surface (Figure 5E). This produced the apparent decrease in size observed in the placebo-treated mice after d 10. Treatment with AZ10606120 significantly inhibited the kinetic of tumor growth, leading to a 50% reduction of tumor size at the time of excision (Figure 5A–5C), thus confirming the strong growth inhibitory effect observed *in vitro*. Size of a representative tumor mass (placebo to the left) is shown in the inset in Figure 5C. IST-MES2 tumor masses were much smaller than MSTO-211H (tumor volume at d 6 post inoculum was 8–10 and 40–50 mm^3^ for IST-MES2 and MSTO-211H, respectively). Intra-mass administration of placebo (started at d 6) caused a growth arrest lasting up to d 12 (Figure 5B). At this time-point the placebo-treated tumor masses started to regress, shrinking to a 2–3 mm size by d 15. In the animals treated with AZ10606120 tumor regression was significantly accelerated, starting soon after drug administration. At d 15, size of excised masses from placebo- or AZ10606120-treated mice was not significantly different. IST-MES2 masses are reported as weight since small size did not allow reliable volume measurement (Figure 5D).

**Figure 6:**
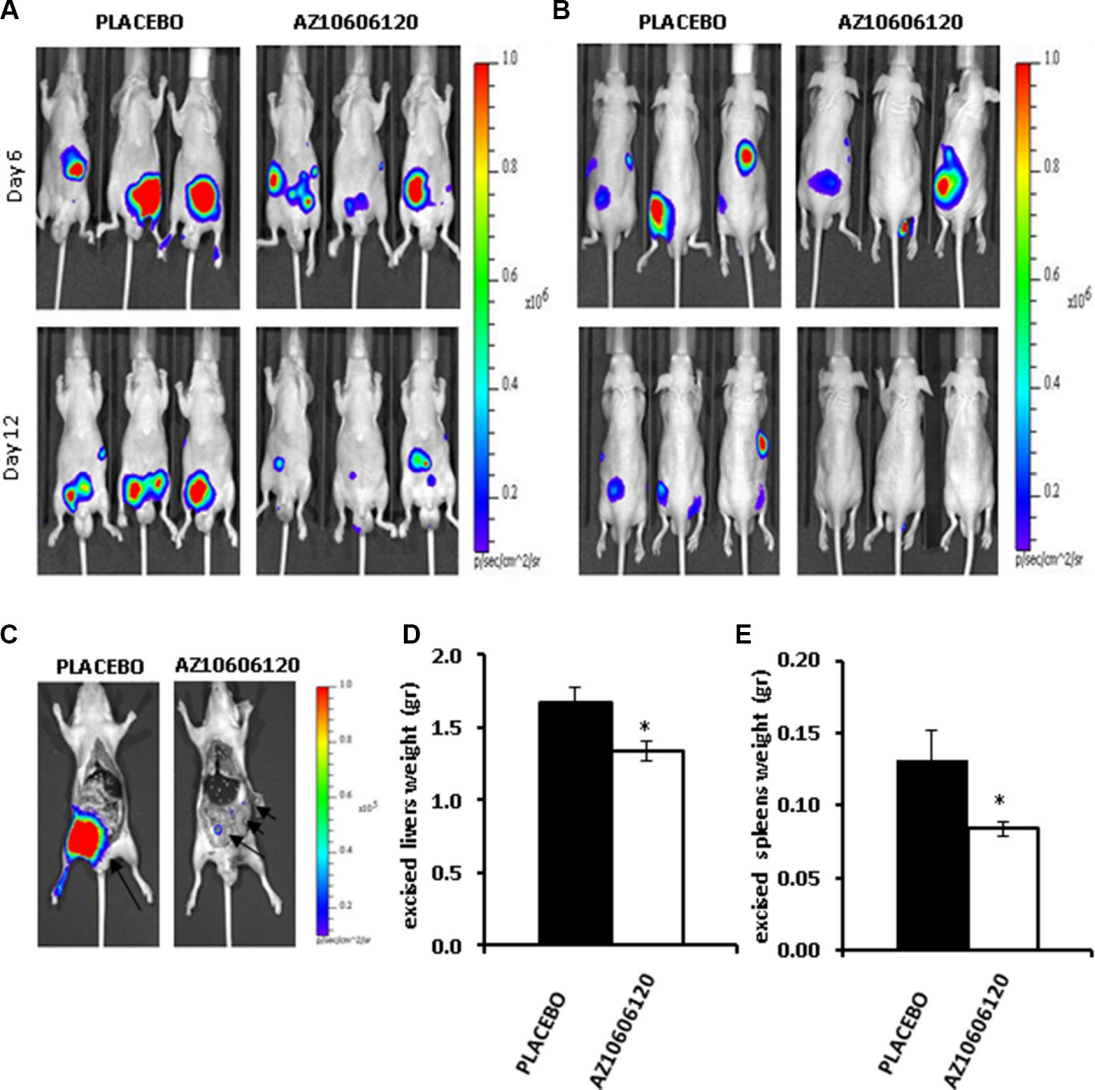
P2×7R-targeting inhibits growth of i.p. inoculated MPM cells 2 × 10^6^ MPP89/CytLUC cells were i.p. inoculated as described in Materials and Methods and on post-inoculum d 6 AZ10606120 (0.7 mg/kg), or placebo (DMSO 0,05% in 100 μl PBS solution) were administered i.p. every two d for up to 12 d. Three mice were used for AZ10606120 and placebo administration, respectively. Lumiscence emission was recorded at post-inoculum d 6 and 12 from ventral (**A**) and dorsal (**B**) projection. (**C**) Representative picture showing luminescence emission from the peritoneum of two mice respectively treated with placebo or AZ10606120 and sacrificed at d 12. Weight of excised livers (**D**) and spleen (**E**) from placebo and AZ10606120-treated mice. Data are averages ± SE; *n* = 3, *p < 0.05.

Tumor cell inoculation in the s.c. tissue of the hip does not faithfully reproduce the physiological environment of mesothelioma growth, therefore we investigated the effect of P2×7R targeting in MPM cells growing in the peritoneal cavity, an anatomical site where 10–20% of human mesotheliomas are commonly found [28]. To this aim we choose the MPP89 cells, which in *in vitro* experiments appeared to be the least sensitive to P2×7R blockade, the rationale being that should P2×7R blockade be effective also under such unfavorable *in vivo* conditions, this would strongly validate P2×7R targeting as a viable approach to fight mesothelioma. In order to follow tumor growth within the peritoneum, an anatomical site that is not amenable to measurement by caliper, MPP89 cells were stably transfected with a cytosolic luciferase construct (MPP89-cytLUC cells) and their growth was monitored by *in vivo* imaging with a total body IVIS luminometer. Mice were i.p. inoculated with the MPP89-cytLUC cells, and treated with AZ10606120 or placebo i.p. administered every two d after tumor mass became first detectable (post inoculum d 6), for a total of 12 d (Figure 6A–6B). At variance with mice injected subcutaneously, i.p.-inoculated, placebo-treated, mice showed clear signs of discomfort (irritability and aggressiveness) as early as post inoculum d 10, thus dictating an early compassionate suppression at d 12. Very interestingly, AZ10606120-treated mice showed no detectable signs of discomfort throughout the treatment. Diffusion of MPP89-cytLUC cells in the peritoneal cavity could be easily followed in both the placebo- and AZ10606120-treated groups up to post inoculum d 8. At later time points in the placebo-treated mice the peritoneal cavity became massively infiltrated with connective tissue, to an extent that luminescence emission was so perturbed to prevent an accurate analysis of tumor cell diffusion. However, despite this perturbing factor, AZ10606120-treated mice showed a more localized and fainter luminescence emission whether recorded from a ventral or a dorsal projection, suggesting a reduced diffusion of the tumor cells in the peritoneal cavity. At post-inoculum d 12, mice were sacrificed and the peritoneal cavity analyzed for luminescence emission. As shown in Figure 6C, while the placebo-treated mouse showed a diffuse infiltration of the peritoneal cavity, only a localized, low intensity, luminescence spot was detectable in the AZ10606120-treated mouse. Examination of the peritoneal cavity also revealed a diffuse connective tissue infiltrate and liver and spleen enlargement in the placebo- but not in the AZ10606120-treated mice (Figure 6D–6E).

## DISCUSSION

Signalling via extracellular ATP has a major role in supporting cell growth and differentiation by activating the P2YRs or P2XRs [23]. P2YRs have been traditionally credited with growth-promoting activity, as they are G- protein-coupled, and thus linked to phosphoinositide hydrolysis, Ca^2+^ mobilization from intracellular stores and activation of the MAPK pathway. More recent studies showed that the P2×7R also supports cell proliferation [13, 26, 29]. This was unanticipated due to the well-known cytotoxic effect due to activation of the P2×7R-associated large conductance pore [30, 31]. It is now clear that under physiological conditions, i.e. under stimulation by locally-released ATP, the P2×7R promotes a trophic response, without the untoward effects due to indiscriminate large pore opening. We have extensively characterized the mechanism responsible for the trophic P2×7R activity identifying the mitochondria as a key organelle [16, 32]. Tonic, low intensity, P2×7R activation stabilizes the mitochondrial network, and increases mitochondrial membrane potential, the Ca^2+^ content of the mitochondrial matrix and the overall efficiency of the oxidative phosphorylation, with the end result of increasing the total cellular ATP content. P2×7R expression also stimulates the Warburg effect, increasing glycolytic ATP generation as well as availability of carbon species for anapleurotic processes [17]. Thus, it appears that P2×7R enhances ATP generation via both the mitochondrial and glycolytic pathways, and in addition increases availability of building blocks for synthetic processes. Besides fueling energy metabolism, P2×7R also increases invasiveness and metastatic ability of cancer cells [9]. Given these P2×7R activities, it is not surprising that most, although but by no means all, malignant tumors so far tested overexpress P2×7R.

Malignant pleural mesothelioma is a peculiar tumor due to its very slow growth rate and very long latency. Therefore, on the basis of the its known growth-promoting activity we hypothesized that the P2×7R should be expressed to a low level. Our findings support this anticipation, and at the same time confirm that P2×7R, despite its low expression level, confers to MPM cells a growth advantage. In fact, HMCs kept under conditions of limited nutrient supply have a very slow growth rate over a 72 incubation time, and accordingly express little P2×7R, whether at the protein or mRNA level. However, immortalization of HMCs with the SV 40 large T antigen increases both growth rate and P2×7R expression. The three MPM cell lines investigated in the present work express P2×7R to a variable, but always very low level, and P2×7R-dependent responses are very small. Nevertheless, P2×7R inhibition by selective blockers or overstimulation by the pharmacological agonist (BzATP) inhibits growth, hinting to a growth-promoting activity of this receptor in MPM cells. While tonic P2×7R stimulation supports growth, its pharmacologic stimulation triggers cytotoxicity. Sensitivity to ATP-mediated cytotoxicity is largely dependent on the level of P2×7R expression, thus it is not surprising that MPM cell lines showed little sensitivity to P2×7R-dependent cytotoxicity *in vitro*. Rather, our data suggest that P2×7R pharmacological stimulation, or conversely inhibition, promote a cytostatic effect.

As it is well known, MPM is highly refractory to chemotherapy, probably due to its inherent slow growth rate. Therefore we asked if P2×7R targeting might be effective to slow down *in vivo* MPM growth. We tested the effect of the highly selective P2×7R drug-like antagonist AZ10606120 in three different *in vivo* settings: nude/nude mice inoculated s.c. with MSTO-211H or IST-MES2 cells, or i.p. with MPP89 cells. The i.p. route was investigated because we thought that it might better mimic the physiological site of MPM growth. In both cases we observed a large reduction of tumor growth in the AZ10606120-treated host. Kinetics of tumor growth in the peritoneum was of particular interest because here MPM cells diffusely infiltrated both visceral and parietal peritoneal layers causing at the same time a visible discomfort to the host. On the other hand, in the AZ10606120-treated animals there was no infiltration at all of the peritoneal layers, and the animal showed no signs of discomfort. Very few therapies are currently available to treat malignant mesothelioma, with a near 100% death rate within one year from diagnosis. Several P2×7R drug like antagonists have been extensively tested for their safety and efficacy for the treatment of chronic inflammatory diseases in over 20 PhaseII/III clinical trials [33]. Our findings might open the avenue for an exploratory trial to test the efficacy of P2×7R blockers in the therapy of malignant pleural mesothelioma.

## MATERIALS AND METHODS

### Cell culture and transfections

HMCs were obtained from biopsies collected from young, non-oncologic patients (age 29–35 years) affected by pneumothorax, and propagated as primary cultures in RPMI 1640 medium (EuroClone SpA, Milano, Italy) supplemented with 15% fetal bovine serum (Thermo Fisher Scientific, Waltham, MA, USA). MPM cell lines, MPP89, MSTO-211H and IST-MES2 from our laboratories [34], were grown in DMEM, supplemented with 2 mM L-Glutamine, 100 U/ml penicillin and 100 mg/ml streptomycin, 10% FBS (EuroClone). Cells were maintained at 37°C in a 5% CO_2_-humidified atmosphere (34). The immortalized mesothelial cell line SV40-Tag was obtained by transfecting HMCs with the pSV3neo plasmid carrying the Simian Virus 40 (SV40) T-antigen (Tag) (Quiagen, Chatsworth, CA). Single-cell–derived clones were obtained by limiting dilution and kept under selection in the presence of 100 μg/ml G418-sulfate (Calbiochem, La Jolla, CA, USA) [35]. For *in vivo* experiments MPP89 cells were transfected with a cytosolic Luciferase (CytLUC) inserted in a pGL4.50 plasmid (Promega Italia, Milan, Italy). A Mirus kit (Mirus Bio LLC, Madison, WI, USA) was used for transfection. Single-cell-derived clones were selected in the presence of 100 μg/ml hygromycin (Sigma-Aldrich, Milano, Italy). MSTO-211H, IST-MES2 and SV40-Tag cells were cultured in RPMI medium supplemented with 15% fetal calf serum, 100 U/ml penicillin and 100 mg/ml streptomycin. The SV40-Tag culture medium was also supplemented with 100 μg/ml G418-sulfate (Calbiochem). MPP89 and MPP89/CytLUC cells were cultured in DMEM-F12 (Sigma-Aldrich) supplemented with 15% fetal calf serum, 100 μg/ml hygromycin Sigma-Aldrich, only MPP89/CytLUC cells), 100 U/ml penicillin and 100 mg/ml streptomycin.

### Cell stimulation and reagents

All experiments were performed in the absence of serum (i.e. serum-free RPMI or DMEM-F12). Cells were plated overnight in complete medium, to be replaced with serum- free medium for the next 72 hours. The P2×7R inhibitor AZ10606120 (Tocris Bioscience, Ellisville, MS, USA) was dissolved as a stock solution of 100 mM in DMSO, and used for all *in vitro* and *in vivo* experiments at a final concentration of 300 nM in sterile PBS (EuroClone).

### Measurement of lactate dehydrogenase release

Cells (5 × 10^5^) were suspended in complete medium and over-night plated in 24-well plates. Incubation medium was then replaced with serum free RPMI or DMEM-F12 supplemented with the different stimulants. At the indicated time points supernatants were collected, cleared by centrifugation (10 min at 250 g) and LDH content determined by spectrophotometry as previously described [36]. Samples lysis with 0.1% Triton ×-100 provided total LDH cell content (100% LDH release).

### Cell proliferation assay and microscopy

Cell proliferation was assessed by crystal violet staining. Cells were seeded over-night in 12-well plates in complete medium at the concentration of 5 × 10^4^ / well. Incubation medium was then replaced with serum free RPMI or DMEM-F12 at 37°C, up to 72 hours. Plates were stained with crystal violet (0.1% in distilled H_2_O) at each indicated time point. Crystal violet was extracted with 10% acetic acid followed by plate reading at 590 nm. Each experiment was performed in triplicate. Proliferation rate was expressed as fold increase over time zero. Pictures were taken every 24 hours with a Leica DMIL LED phase contrast microscope equipped with a 10× objective and a color camera (Leica Microsystems, Germany).

### RT-PCR and qRT-PCR

Total mRNA was extracted with TRIzol Reagent and the PureLink RNA Mini Kit (Thermo Fisher Scientific), according to manufacturers' instructions. The PCR products were analyzed on 1.2% agarose gel implemented with Gel Red dye (Thermo Fisher Scientific), and revealed with Gel Red Imaging System Documentation equipment (Alpha Innotech, San Leandro, CA, USA). qRT-PCR was performed with the High-Capacity cDNA Reverse Transcription Kits (Applied Biosystems, Foster City, CA, USA). Samples were run in triplicate in an AB StepOne Real Time PCR (Applied Biosystems) with TaqMan Gene Expression Master Mix (Applied Biosystems) using the following TaqMan probes: NM_002562.5 (P2×7R, Life Technologies) and NM_001101.2 (human β-actin, Applied Biosystems).

### Western blotting

Cells were detached by scraping and lysed in lysis buffer (300 μM sucrose, 1 mM K_2_HPO_4_, 1 mM MgSO_4_, 5.5 mM glucose, 20 mM HEPES (pH 7.4), 1 mM benzamidine, 1 mM phenylmethylsulfonyl fluoride, 0.2 μg DNase, and 0.3 μg RNase, all by Sigma-Aldrich) by repeated freeze/thawing cycles. Proteins were separated on 8% Bolt-SDS precast gels (Thermo Fisher Scientific) and blotted on nitrocellulose paper (GE Healthcare Life Science). Membranes were incubated overnight with the anti-P2×7 antibody (Sigma-Aldrich), at a dilution of 1:200 in Tris-buffered saline (TBS: 50 mM Tris, 150 mM NaCl, pH 7.6), supplemented with 3% non-fat milk and 0.5% bovine serum albumin (BSA). The anti-β actin Ab was used at a dilution of 1:1000 in TBS plus 5% BSA. The secondary anti-rabbit horseradish peroxidase-conjugated Ab (Dako, Milan, Italy) was used at a dilution of 1:2000 in TBS-t buffer (TBS, 0.1% Tween-20, Sigma-Aldrich). Protein bands were revealed with an enhanced chemiluminescence detection kit (GE Healthcare Life Science).

### Intracellular free Ca^2+^ concentration measurement

Changes in the cytoplasmic Ca^2+^ concentration were measured with the fluorescent indicator fura-2-acetoxymethyl ester (fura-2/AM, 4 μM) (Thermo Fisher Scientific) in a thermostat-controlled (37°C) and magnetically stirred Cary Eclipse Fluorescence Spectrophotometer (Agilent Technologies, Milan, Italy). For fura-2-AM loading cells procedure, 10^6^/mL cells were incubated for 20 minutes in a saline solution containing 125 mM NaCl, 5 mM KCI, 1 mM MgSO_4_, 1 mM Na_2_HPO4, 5.5 mM glucose, 5 mM NaHCO3, 1 mM CaC1_2_ and 20 mM Hepes (pH 7.4), as previously described and heretofore referred to as standard saline [37]. Measurements were carried out at the 340/380 nm excitation wavelength ratio and at an emission of 505 nm, then converted into intracellular nano-molar Ca^2+^ concentration by applying the following general formula:

[Ca^2+^]_i_ = K_d_(R – R_min_)/(R_max_ – R) (F_o_/F_s_), where Kd is 224 nM, R is the 340:380 ratio of fluorescence of the intracellular indicator, R_min_ is the 340:380 ratio of fura-2 at 1 nM Ca^2+^, R_max_ is the 340:380 ratio of fura-2 fluorescence intensity in the presence of saturating Ca^2+^ concentrations, and F_o_/F_s_ is the ratio of fura-2 fluorescence emission at 1 nM and at saturating Ca^2+^ concentrations at excitation 380 nm.

### Pore-forming activity

P2×7-dependent pore formation and increases in plasma membrane permeability were measured by monitoring uptake of the fluorescent dye ethidium bromide (Sigma-Aldrich), as previously described (37). Briefly, cells (5 × 10^5^/ml) were incubated in a standard saline solution without CaCl_2_, plus 1 mM EGTA and 20 μM ethidium bromide, in a thermostat-controlled (37°C) fluorometric cuvette under constant magnetic stirring. Uptake of the fluorescent dye was monitored over 25 min. Digitonin (100 μM) was added to achieve complete permeabilization of cells (100% fluorescence signal). Fluorescence was measured at 360 nm/580 nm excitation/emission wavelengths, with a Cary Eclipse Fluorescence Spectrophotometer (Agilent Technologies).

### Analysis of *in vivo* tumor growth

*Nude/nude* mice were purchased from Harlan Laboratories (Harlan Italy, S. Pietro al Natisone, Italy). 2 × 10^6^ human mesothelioma MSTO-211H or IST-MES2 cells were (s.c.) injected into four week-old male mice. Tumor masses became detectable four d after injection. Mice were split into two groups and either treated with placebo (PBS supplemented with 0.05% DMSO) or the P2×7R inhibitor AZ10606120 (300 nM, Tocris Bioscence), administered intra-mass every two d. Tumor mass was measured every 2 d by micro-caliper. Twenty d (MSTO-211H) or 15 d (IST-MES2) after tumor inoculum mice were sacrified and masses explanted. Tumor volume was calculated with the following equation: volume = π*/*6 [w1 × (w2)^2^], where w1 = major diameter and w2 = minor diameter (13). In a second run of experiments, 2 × 10^6^ human mesothelioma MPP89 cells stably transfected with cytoplasmic luciferase (MPP89/CytLuc cells) were (i.p.) injected into four week-old male mice. Four d after the injection, tumor masses became detectable by luminescence analysis (IVIS Lumina, Caliper-Perkin Elmer, Hopkinton, MA, USA). Thereafter, animals were split into two groups and treated with either placebo (PBS supplemented with 0.05% DMSO) or the P2×7R inhibitor AZ10606120 i.p. administered every two d. Tumor growth was monitored every 2 d by luminescence analysis. Twelve d after inoculum mice were sacrified, and livers and spleens excised and weighed. Prior to luminescence emission acquisition, mice were anesthetized (1–3% isofluorane) and i.p. injected with luciferin (150 mg/kg) (Perlin-Elmer). Luminescence emission from regions of interest was quantified by photon counting and image analysis using the Living ImageH software (Xenogen Corp, Alameda, CA). Luminescence emission was also acquired from the open peritoneal cavity of sacrified animals by directly perfusing the open abdominal cavity with D-luciferin. Animal procedures were approved by the University of Ferrara Ethic Committee and the Italian Ministry of Health in compliance with international laws and policies (European Economic Community Council Directive 86/109, OJL 358, Dec. 1, 1987, and NIH Guide for the Care and Use of Laboratory Animals).

### Data analysis

All data are shown as mean ± S.E. Tests of significance were performed by unpaired *t*-test with Welch's correction, using GraphPad InStat software (GraphPad, San Diego, CA, USA).
